# Virtual obstetrics and gynecology fellowship interviews during the coronavirus disease 2019 (COVID-19) pandemic: a survey study

**DOI:** 10.1186/s12909-021-02893-4

**Published:** 2021-08-26

**Authors:** Alexandra Peyser, Moti Gulersen, Michael Nimaroff, Christine Mullin, Randi H. Goldman

**Affiliations:** 1 Division of Reproductive Endocrinology and Infertility, Department of Obstetrics and Gynecology, North Shore University Hospital – Zucker School of Medicine at Hofstra/Northwell, Manhasset, NY USA; 2 Division of Maternal-Fetal Medicine, Department of Obstetrics and Gynecology, North Shore University Hospital – Zucker School of Medicine at Hofstra/Northwell, Manhasset, NY USA; 3 Division of Minimally Invasive Gynecologic Surgery, Department of Obstetrics and Gynecology, North Shore University Hospital – Zucker School of Medicine at Hofstra/Northwell, Manhasset, NY USA

**Keywords:** Virtual interviews, Obgyn, Fellowship, COVID-19

## Abstract

**Background:**

Due to the coronavirus disease 2019 (COVID-19) pandemic, all Obstetrics and Gynecology fellowship interviews were held virtually for the 2020 fellowship match cycle. The aim of this study was to describe our initial experience with virtual Obstetrics and Gynecology fellowship interviews and evaluate its effectiveness in assessing candidates.

**Methods:**

This was a cross-sectional survey study that included all interviewing attending physicians and fellows from five Obstetrics and Gynecology subspecialties at a single academic institution following the 2020–2021 fellowship interview season. The survey consisted of 19 questions aimed to evaluate each subspecialty’s virtual interview process, including its feasibility and performance in evaluating applicants. The primary outcome was the subjective utility of virtual interviews. Secondary outcomes included a comparison of responses from fellows and attending physicians.

**Results:**

Thirty-six attendings and fellows completed the survey (36/53, 68% response rate). Interviewers felt applicants were able to convey themselves adequately during the virtual interview (92%) and the majority (70%) agreed that virtual interviews should be offered in future years. Attending physicians were more likely than fellows to state that the virtual interview process adequately assessed the candidates (Likert Scale Mean: 4.4 vs. 3.8, respectively, *p* = 0.02). Respondents highlighted decreased cost, time saved, and increased flexibility as benefits to the virtual interview process.

**Conclusion:**

The use of virtual interviews provides a favorable method for conducting fellowship interviews and should be considered for use in future application cycles. Most respondents were satisfied with the virtual interview process and found they were an effective tool for evaluating applicants.

**Supplementary Information:**

The online version contains supplementary material available at 10.1186/s12909-021-02893-4.

## Introduction

The coronavirus disease 2019 (COVID-19) pandemic has compelled graduate medical education (GME) programs to conduct residency and fellowship interviews virtually for applicants given social distancing requirements and global travel restrictions. Traditionally, applicants apply for their selected fellowship through the Electronic Residency Application Services (ERAS) and selected applicants are invited by programs for a formal in-person interview. The transition from in-person to virtual interviews was supported by the May 2020 statement from the Association of American Medical Colleges, which strongly encouraged interviews for medical school, residency, and faculty applicants to be conducted using virtual platforms [1]. Subsequently, Obstetrics and Gynecology (Ob/Gyn) fellowship programs across the United States initiated virtual interviews for the 2020 interview season. Prior to the ongoing public health crisis, a few institutions had utilized virtual interviewing, however, the majority transitioned back to in-person interviews [2–4].

Several studies have examined the complete transition to virtual interviews during the pandemic for training programs from a variety of medical specialties [5–11]. As remote interviews may become commonplace following resolution of the pandemic, it is important to evaluate both programs’ and applicants’ perspectives on using virtual platforms. For applicants, virtual interviews may alleviate some of the large economic burden of travel and loss of clinical time from residency. However, applicants may not be able to showcase their personalities as well virtually as they would in-person. Whereas programs, on the other hand, may not be able to portray their attributes to the fullest potential in a virtual setting.

The purposes of this study were to survey attending and fellow physician interviewers across five Ob/Gyn subspecialties (Maternal Fetal Medicine [MFM], Gynecologic Oncology [Gyn-Onc], Reproductive Endocrinology and Infertility [REI], Minimally Invasive Gynecology [MIGS] and Female Pelvic Medicine and Reconstructive Surgery [FPMRS] and describe their experiences with virtual Ob/Gyn fellowship interviews for the 2020 cycle, and to determine feasibility for future use.

## Methods

This was a cross-sectional survey study including all interviewing attending physicians and fellows from five Obstetrics and Gynecology subspecialties at a single academic health institution following the 2020–2021 fellowship interview season. An anonymous 19-question survey was created using Northwell’s Research Electronic Data Capture Tool (REDCap) and distributed via email 3 times between October 20, 2020 and November 29, 2020, after the completion of interviews for the 2020–2021 fellowship year. Survey links and responses did not carry any identifiers to ensure confidentiality and anonymity.

Study data were collected and managed using REDCap electronic data capture tools hosted at Northwell Health. REDCap is a secure, web-based application designed to support data capture for research studies, providing (1) an intuitive interface for validated data entry; (2) audit trails for tracking data manipulation and export procedures; (3) automated export procedures for seamless data downloads to common statistical packages; and (4) procedures for importing data from external sources.

The list of all attendings and fellows who participated in interviews was provided to the authors by each subspecialty training manager. A total of 53 eligible physicians were identified, including 35 attendings and 18 fellows. The questionnaire was created by two fellows (AP, MG) and an associate program director (RG), and consisted of 19 questions requiring approximately 2 min to complete (Additional file 1). The survey was reviewed for clarity, ease of use and functionality by members of the research team and several faculty members prior to being more widely distributed. Survey questions were adapted from previous studies in other fields of medicine (Robinson et al. and Majumder et al.) [6, 7].

The survey assessed objective components of the interview including the platform used, how the program was introduced, the format of the interview (i.e how many attendings to applicants), if fellows interviewed applicants separately, as well as subjective measures including perspectives on the use of virtual interviews, the adequacy of evaluation of applicants, and whether they felt applicants should perform a mock virtual interview in advance. It also addressed perceived advantages and disadvantages to virtual interviews. Opportunities for free response answers were included as well.

A 5-point Likert scale was used to assess perceptions regarding the ease of use and satisfaction with the virtual interview process (“strongly agree [5], agree, neither agree nor disagree, disagree and strongly disagree [1]). Descriptive statistics were used for analysis of the cohort’s characteristics. Likert scales were converted to numerical scores (1–5) in order to compare differences in responses between attendings versus fellows. The Mann-Whitney test was used to compare mean scores between attendings and fellows with statistical significance set as *p* < 0.05.

## Results

A total of 36 surveys were completed yielding a 68% response rate (36/53). Survey respondents were representative of all subspecialties eligible for this study (27% MFM, 25% REI, 19% MIGS, 14% FPMRS, and 14% Gyn-Onc, Table 1).

**Table 1:** Respondent demographics

Department	n (%)
MFM	10 (28)
REI	9 (25)
Gyn Onc	5 (14)
MIGS	7 (19)
FPMRS	5 (14)
**Current Position**	
Fellow	18 (50)
Attending	18 (50)

Fifty percent of respondents were attending physicians and the other half were fellows. All specialties utilized Zoom® (Zoom Video Communications, San Jose, California, USA) as their virtual platform. Ninety two percent reported no technical difficulties during the interview process.

Interview format varied based on subspecialty (Table 2). Two specialties had 2 attendings and 1 applicant in a session (REI, Gyn Onc), 1 specialty had 3 attendings to 1 applicant (MIGS), and 1 specialty had 1 attending to 1 applicant (MFM). FPMRS interview sessions were varied. All specialties had fellows meet separately with the applicants with no attendings present; however, the structure of fellow interviews between specialties differed with either a question and answer session with the fellows and multiple applicants (REI, MIGS, Gyn Onc) or a formal interview with all fellows and one applicant (MFM, FPMRS). Interviews lasted approximately 15–20 min (60%) or 30 min (40%). MFM, Gyn Onc and MIGS had no breaks between interviews, whereas REI and FPMRS had a 5–10 min break between interviews.

**Table 2:** Interview format by specialty

Specialty	Duration of each interview	Duration of break between interviews	Attending to applicant interviewing	Fellow interviews	Virtual tour	How the program was introduced
REI	15–20 min	5–10 min	2 to 1	Yes	Yes	Recorded Video and Live in Person By Attending
MFM	30 min	None	1 on 1	Yes	No	Powerpoint and Live in Person by Attending
Gyn-Onc	30 min	None	2 to 1	Yes	No	Live in Person By Attending
FPMRS	15–20 min	5–10 min	Varied	Yes	No	Powerpoint and Live in Person by Attending
MIGS	15–20 min	None	3 to 1	Yes	No	Recorded Video and Live in Person by Attending

When introducing the fellowship program, two of the programs utilized a recorded video and live in-person introduction by an attending, two used Powerpoint® (Microsoft Corporation, Redmond, Washington, USA) and a live in-person introduction, and one used just a live in-person introduction. A virtual tour was given only by the REI division.

Attending and fellow respondents felt that the most important factor in evaluating the overall quality of applicants was the virtual interview (66%), followed by direct conversations with attendings at the applicants’ institutions (14%). The written application and letters of recommendation were felt to be less important (11 and 9%, respectively).

**Fig. 1:**
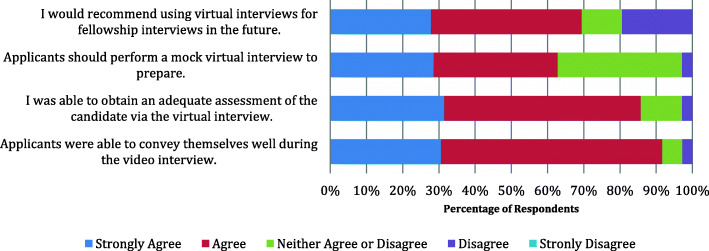
Responses on virtual interview use for fellowship interviews

Interviewers believed applicants were able to convey themselves well during the virtual interview (31% Strongly Agreed, 61% Agreed; Likert scale mean: 4.2). Most respondents felt that they were able to obtain an adequate assessment of the candidate during the interview process (31% Strongly Agreed, 54% Agreed, Likert scale mean: 4.1) with 3% disagreeing. The majority of participants agreed that they would recommend virtual interviews for future use (28% Strongly Agreed, 42% Agreed, Likert scale mean: 3.7), while 19% disagreed. Most respondents agreed that applicants should prepare with a mock interview prior to the virtual interview (29% Strongly Agree, 34% Agree, Likert scale mean 3.8) (Fig. 1).

**Fig. 2:**
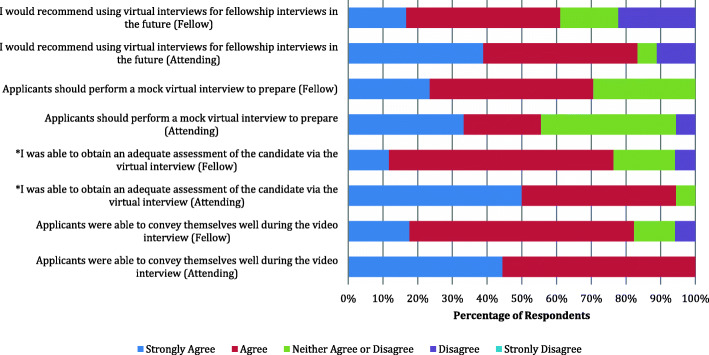
Attending and fellow responses to evaluation of applicants, performing mock interviews, and recommendation for future use. * denotes significance

When comparing responses between attendings and fellows, attendings were more likely than fellows to report that they could assess candidates as a whole via the virtual platform (4.4 vs. 3.8, respectively, *p* = 0.02). There was no difference between attending and fellow responses in regard to applicants adequately conveying themselves during the virtual interview (4.4 vs. 3.9, respectively, *p* = 0.06) and no difference in the recommendation of utilizing virtual interviews in the future (4.0 vs. 3.5, respectively, *p* = 0.20) (Fig. 2).

Eighty-eight percent of respondents agreed that financial savings for applicants was the biggest advantage of virtual interviews, followed by time saved for applicants (66%), and increased efficiency for departmental staff (36%) (more than one answer could be chosen). Respondents also agreed that the biggest disadvantage of virtual interviews included less social interaction with applicants (66%) and no facility tour (45%). Other disadvantages in free response answers included no in-person discussion of applicants following the interview days and difficulty assessing applicants virtually. There were no applicant complaints about the virtual interview platform for any subspecialty. Free response answers regarding how interviews can be enhanced in the future included adding a virtual tour, practice interviewing virtually, and improving online resources available to applicants (Table 3).

**Table 3:** Free responses to “How do you think virtual interviews can be improved in the future?”

Should be used permanently.	
Make them shorter.	
Virtual tour of the facilities.	
More practice interviewing virtually.	
I would recommend an end of the day group send off by the fellowship director to all applicants for closing remarks and any last minute questions from applicants.	
It was a seamless process, worked great. A virtual tour and may be some way to have some social time on Zoom will be helpful.	
More online information available to applicant.	
Providing virtual tours.	
Add a virtual tour of the facility.	
By not occurring.	
I think they will improve as we all get more comfortable with the virtual environment through repeated use.	
A video of the facilities and surrounding community.	
Better format and include virtual tour.	

## Discussion

Due to the COVID-19 pandemic, all Ob/Gyn fellowship programs across the United States transitioned to a virtual interview platform. Our study demonstrated that virtual interviews were effective, as a majority of interviewers felt that they were able to adequately assess candidates via the remote platform. In addition, most recommended future use of virtual interviews. We believe the lessons learned from this experience will be valuable in providing guidance to the planning processes for virtual interviews, should they continue in future application years.

All programs utilized Zoom as the platform, with no technical issues reported. Program introductions were varied, with most utilizing either live in-person introductions by an attending, a Powerpoint, and/or a pre-recorded video. In previous studies that have analyzed the utility of web-based interviews, several strategies were used to introduce the program and promote engagement with faculty. These included: a prerecorded video by the program director (PD), a live stream session with the PD, or a hybrid approach [11, 12]. The live-streamed session has been shown to offer greater exchange of information and interaction with the program director [5]. In a recent study analyzing cardiothoracic fellowship applicants and program directors’ views on the virtual interview process during the pandemic, applicants felt that introductions should be an open interaction or a private session with trainees to give as much exposure to faculty and staff as in-person interviews otherwise would [6].

Virtual interviews offer several advantages over traditional in-person interviews including greater convenience, lower costs for applicants and programs, increased scheduling flexibility, and mitigation of geographic constraints [6]. Additionally, applicants are able to apply to a greater number of programs given the travel cost savings. On average, residents applying to fellowship spend on average $4000–$8000 on the fellowship interview process and miss an average of 7–10 days of clinical work for travel [13]. Programs may also have a financial burden, spending an average of $8000 per fellowship cycle (including food, social gatherings, supplies, shuttle fees). Additional estimated effort cost for faculty was $77,000 [14]. In the era of an ever-increasing national student debt, the cost savings associated with virtual interviews also breaks access barriers associated with applicants’ personal financial constraints. Hence, Ob/Gyn fellowship programs should consider restructuring recruitment strategies by including virtual interviews in future application cycles.

Virtual interviews may also pose significant challenges to both applicants and programs. Limitations to remote interviews from programs’ perspectives include missing out on intangible indicators that would otherwise be apparent, such as body language and applicant-to-applicant interactions. From the applicants’ perspective, they may not be able to fully appreciate the relationships between current faculty and fellows, nor have a true understanding of where they will be training and residing due to lack of adequate facility tour. To overcome this, studies have demonstrated that providing applicants with virtual hospital tours, videos, and electronic handouts to be beneficial for applicants [15]. However, in our study the only virtual tour was performed by the REI department. Technical difficulties may also present as a problem, even though in our study, there were no reported technical issues.

Our study found that attendings were more likely than fellows to report that they could assess candidates as a whole via the virtual platform. This finding could be due to attendings having more years of experience with the interview process than fellows in general or that a large amount of fellows participated in a question and answer session as opposed to a one-on-one interview with less interaction with the applicants. Fellow assessment of their future co-fellow is important, and whether virtual interviews hinders this should be explored in future studies.

Previous studies on the virtual interview process have demonstrated mixed opinions regarding future use. In a recent article by Majumder et al., the authors describe their experience with the virtual interview process for Advanced Gastrointestinal Minimally Invasive Surgery Fellowship during the 2020 interview cycle [7]. Applicants and interviewers were both surveyed with a 94% satisfaction rate by applicants and 100% of faculty recommending the use of the virtual platform in the future [7]. In another study, 70% of candidates who took part in a virtual interview for an adult reconstruction fellowship felt that it was an appropriate format when compared with in-person interviews [3]. The latter aligns with our study findings where overall, a majority of respondents felt that virtual interviews should be used in the future.

While in-person interviews have strong benefits, they are not possible in the setting of a public health crisis where the risk of exposure to an infectious disease outweighs the benefits. However, when it is safe to hold in-person interviews in the future, it is possible that the disadvantages of in-person interviews (high cost etc.) will outweigh the benefits validating the utility of virtual interviews. This remains to be determined and future studies are needed to explore this further.

Strengths to this study include participants containing multiple subspecialties within Ob/Gyn. In addition, all subspecialties were well-represented by both attendings and fellows. Lastly, the 2-min survey format allowed for more convenience for completion and participation as a result.

There are several limitations to this study. First, we did not capture the applicants’ perspectives. As this survey was initially sent after rank lists were made and before the match, it was decided to postpone surveying applicants until after the match. As with all surveys, there is the potential for recall bias from respondents; however, the survey was dispersed shortly following interview completion by all subspecialties to minimize this risk. In addition, our sample size is small as this was from a single institution. However, the number of attendings and fellows from fellowship programs is usually relatively small and not all members from each department conducted interviews. Future studies utilizing a greater number of institutions among various geographical regions are needed before drawing definitive conclusions.

## Conclusion

These unprecedented times have impacted the format of residency and fellowship training program interviews, and the adoption of new interview protocols moving forward may be warranted. Based on our survey results, Ob/Gyn fellowships found virtual interviews efficient with a majority of respondents recommending its future use. As Ob/Gyn fellowship matches become increasingly competitive, virtual interviews may be a key tool to alleviate the burdens of in-person interviews and expand the geographic applicant pool. Lessons learned from programs’ experiences with virtual interviews should continue to be studied to determine if this format may be incorporated into routine practice in the future.

## Supplementary Information

**Additional file 1.**

## Data Availability

The datasets analyzed during the current study are available from the corresponding author on reasonable request.
