# Supplementary material for: Virtual obstetrics and gynecology fellowship interviews during the coronavirus disease 2019 (COVID-19) pandemic: a survey study
Source: BMC Med Educ. 2021 Aug 26;21:449. doi: 10.1186/s12909-021-02893-4 (PMC8387093; doi:10.1186/s12909-021-02893-4)
Supplement: Supplementary file 1 — Additional file 1. [file 12909_2021_2893_MOESM1_ESM.docx]

Virtual OBGYN Fellowship Interviews Survey

1. What sub-specialty do you belong to?

a. MFM

b. REI

c. FPMRS

d. MIGS

e. Gyn-Onc

2. Are you a faculty member or a fellow?

a. Faculty

b. Fellow

3. What platform did you use to conduct interviews?

a. Zoom

b. Microsoft Teams

c. Other

4.Did you have any technical difficulty during the virtual interview process?

a. Yes

b. No

5. Please explain the technical difficulty___________

6. What was the length of each interview?

a. 5-10 minutes

b. 15-20 minutes

c. 30 minutes

d. More than 45 minutes

7. What was the number of faculty that interviewed each applicant in a given session?

a. 1

b. 2

c. 3 or more

d. Varied

8. How much time did you give between interviews?

a. 0 minutes

b. 5-10 minutes

c. 15-20 minutes

d. 30 minutes or more

9. Did you have any breaks during the interview day?

a. Yes

b. No

10. How was the program introduced?

a. Video

b. Powerpoint

c. Both

d. None

e. Other

11. Applicants were able to convey themselves well during the video interview

a. Strongly agree

b. Agree

c. Neither agree or disagree

d. Disagree

e. Strongly Disagree

12. I was able to obtain an adequate assessment of the candidate via the virtual interview.

a. Strongly agree

b. Agree

c. Neither agree or disagree

d. Disagree

e. Strongly Disagree

13. Applicants should perform a mock virtual interview to prepare.

a. Strongly agree

b. Agree

c. Neither agree or disagree

d. Disagree

e. Strongly Disagree

14. I would recommend using virtual interviews for fellowship interviews in the future.

a. Strongly agree

b. Agree

c. Neither agree or disagree

d. Disagree

e. Strongly Disagree

15. The most beneficial part of virtual interviews (you may choose more than one answer):

a. Financial savings for applicants

b. Time saved for applicants

c. Increased efficiency for department staff

d. Other

16. If other, please explain:__________

17.The biggest disadvantage to virtual interviews (you may choose more than one answer):

a. Less social interaction with applicants

b. No facility tour

c. Other

18. If other, please explain:____________

19. Any applicant complaints?

1. Yes
2. No

Please explain:_____________
